# Resection and patch aortoplasty for right renal artery aneurysm through a midline laparotomy

**DOI:** 10.1186/s44215-026-00255-4

**Published:** 2026-04-02

**Authors:** Ryo Nakanishi, Hiroaki Osada, Takehiko Matsuo, Kenji Minatoya

**Affiliations:** Department of Cardiovascular Surgery, Graduate School of Medicine, Kyoto University, 54 Shogoin-Kawaharacho, Sakyo-ku, Kyoto, 606-8507 Japan

**Keywords:** Renal artery aneurysm, Calcified aorta, Intraaortic balloon occlusion, Patch aortoplasty

## Abstract

**Background:**

Renal artery aneurysm (RAA) is a rare but potentially life-threatening vascular pathology. Surgical management depends on the aneurysm’s location and morphology. In cases with extensive aortic calcification or anatomical complexity, standard clamping techniques may be infeasible, requiring alternative hemostatic strategies. We present a unique case of RAA in a patient with Takayasu’s arteritis and diffuse aortic calcification, in whom intra-aortic balloon occlusion and patch aortoplasty were used as a salvage technique during surgical repair.

**Case Presentation:**

An 80-year-old woman with a history of Takayasu’s arteritis was referred for surgical treatment of an enlarging right RAA (55 × 74 mm), incidentally detected on imaging during hospitalization for pneumonia. Preoperative computed tomography (CT) demonstrated diffuse calcification of the entire aorta and an aneurysm located at the origin of the right renal artery. The distal segment of the right renal artery was occluded, and the right kidney could not be identified. Due to anticipated bleeding risk and poor clamping accessibility, cardiopulmonary bypass was established via the right femoral vessels. Multiple intra-aortic balloon occlusion catheters were inserted, though several ruptured intraoperatively. Hemostasis was ultimately achieved by inflating a balloon inserted directly through the operative field and performing a patch closure of the dilated right renal artery ostium. The patient required massive transfusion and temporary renal replacement therapy postoperatively. She was discharged to rehabilitation on postoperative day 49. Postoperative CT confirmed patch integrity, but a localized aortic dissection at the balloon site was observed.

**Conclusions:**

This case illustrated the technical challenges of surgical approaches for RAA in patients with severe aortic calcification due to Takayasu’s arteritis. Intra-aortic balloon occlusion is a useful alternative to conventional clamping but carries a risk of rupture, particularly in calcified aorta. Patch aortoplasty may be considered for elderly patients where graft replacement is not feasible. Preoperative imaging is essential to plan individualized strategies for bleeding control and vascular access in high-risk patients.

## Background

Renal artery aneurysm (RAA) is characterized by a dilated segment of the renal artery, which is an uncommon but potentially life-threatening entity, often detected incidentally [1]. The location and shape of the aneurysm determine the repair strategy. Some cases require aneurysmectomy, angioplastic renal artery closure, or nephrectomy for irreparable renal arteries [2]. Furthermore, severely calcified aortas and large aneurysms make it arduous to clamp or access the arteries, frequently requiring a complex surgical approach. We report a successful surgical repair of a peripherally occluded right RAA through a midline laparotomy in an 80-year-old woman with Takayasu’s arteritis. Ethics review is not required for the implementation of case reports in our institution. The patient gave us her full consent for publication of this case.

## Case presentation

**Fig. 1:**
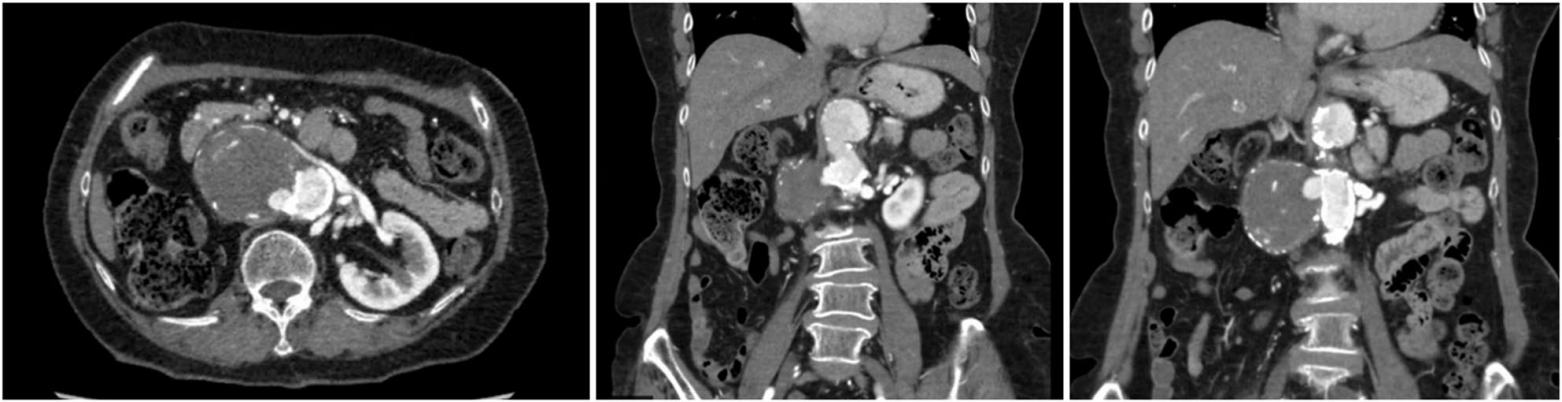
The computed tomography angiography showed the right renal artery aneurysm. Blood inflow is detected inside it from the abdominal aorta. The right kidney is severely atrophic

**Fig. 2:**
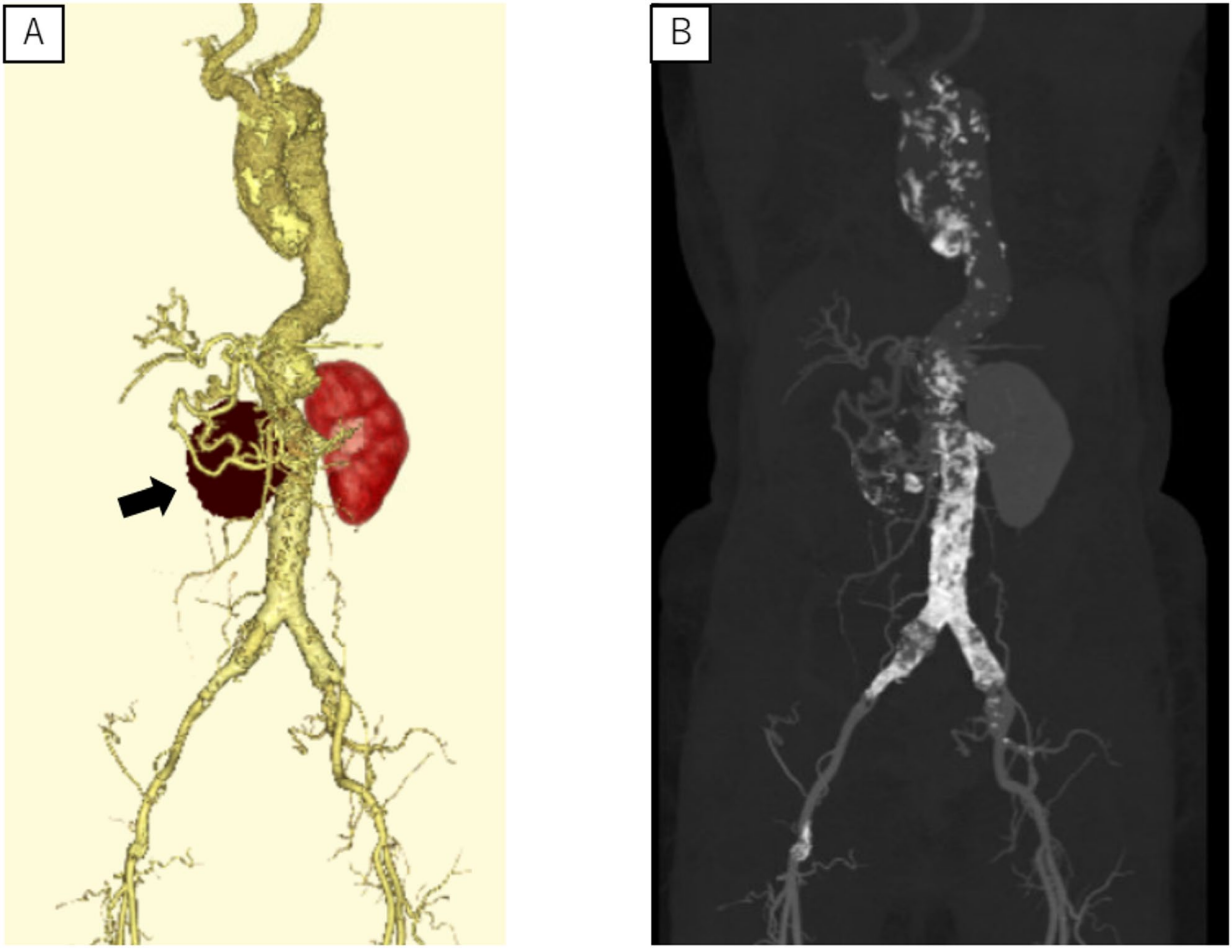
A massive renal artery aneurysm is located on the right side of the abdominal aorta (panel **A**, arrow). The abdominal aorta is highly calcified along its entire length (panel **B**)

An 80-year-old woman with Takayasu’s arteritis since her 30s was diagnosed with a right RAA at age 41 but the RAA was not followed up regularly. She lived alone and was independent in activities of daily living (ADL). She had been receiving low-dose steroid therapy on an outpatient basis. At age 79, during hospitalization for pneumonia, a CT scan showed an enlarged right RAA with peripheral occlusion, leading to referral for surgical intervention. Preoperative CT images revealed a 55 × 74 mm diameter aneurysm at the bifurcation of the right renal artery, with a partial contrast effect inside (Fig. 1). The distal right renal artery was occluded, and the right kidney was not identified in the images (Fig. 2, panel A). The abdominal aorta was highly calcified along its entire length (Fig. 2, panel B). Other cardiovascular complications included right subclavian artery stenosis (99%), left subclavian artery occlusion, right common iliac artery aneurysm (diameter 23 mm), right internal iliac artery occlusion, and significant coronary artery diseases (left anterior descending branch 90% stenosis, and right coronary artery aneurysm (diameter 10 mm) and 99% stenosis). Preoperative percutaneous coronary intervention (PCI) was deferred as the patient was asymptomatic, had a preserved ejection fraction and well-developed collateral arteries. Although the right renal artery could not be visualized, coil embolization was not considered due to the wide and short aneurysmal neck. Therefore, open surgical repair was selected as the treatment strategy.

**Fig. 3:**
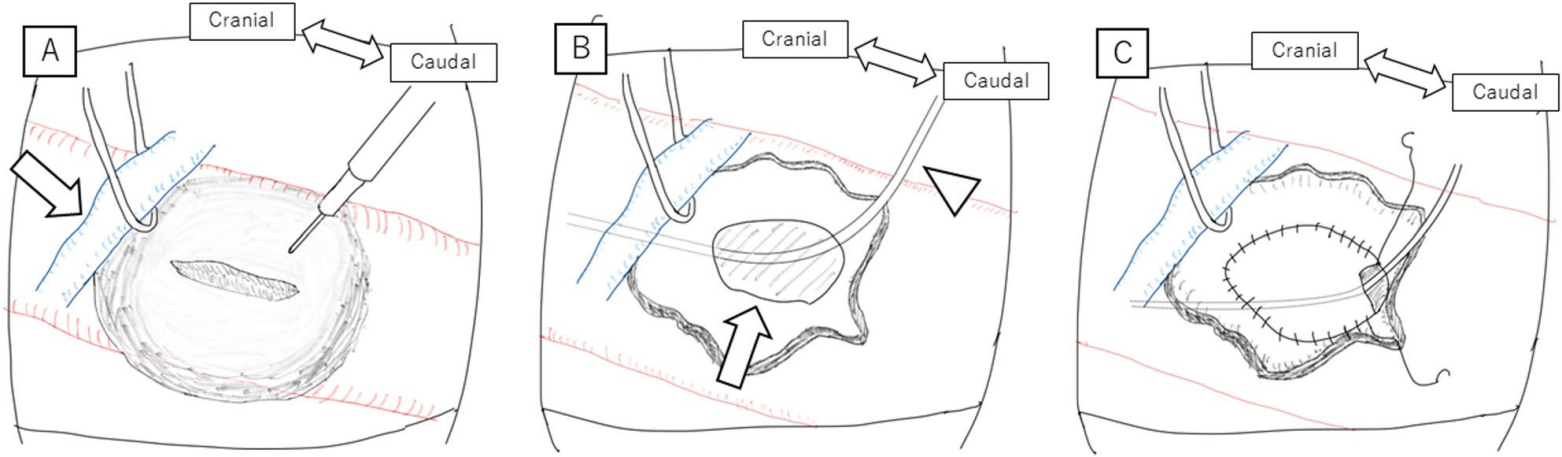
Intraoperative findings. The left renal vein (panel **A**, arrow) is taped with a vessel loop and retracted cephalad to expose the RAA located on the dorsal side. RAA was incised longitudinally (panel **A**). Ostium of the right renal artery was exposed in the aneurysm (panel **B**, arrow), through which MOB balloon was inserted directly (panel B, arrow head). Patch aortoplasty after resection of the aneurysm (panel **C**)

**Fig. 4:**
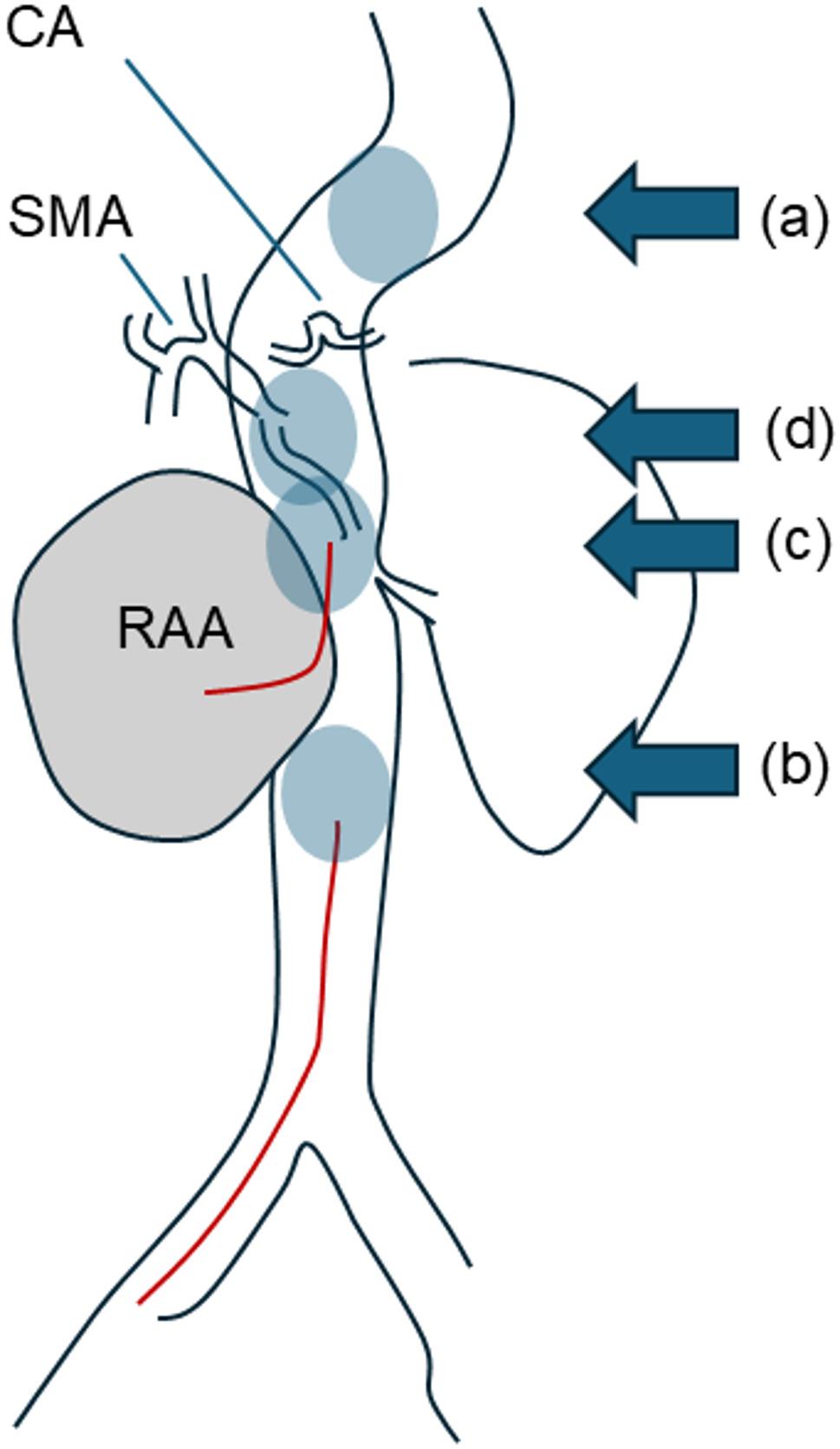
A schematic illustration, clarifying the anatomical relationships and balloon positions. We inserted and placed first two balloons at descending thoracic aorta (**a**) and abdominal aorta (**b**) via right femoral access. Then, next balloon was placed directly above the right renal artery ostium via direct intraoperative insertion (**c**). Additional MOB balloons were placed at SMA ostium (**d**) directly in the operative field, and abdominal aorta (**b**) via the femoral artery

**Fig. 5:**
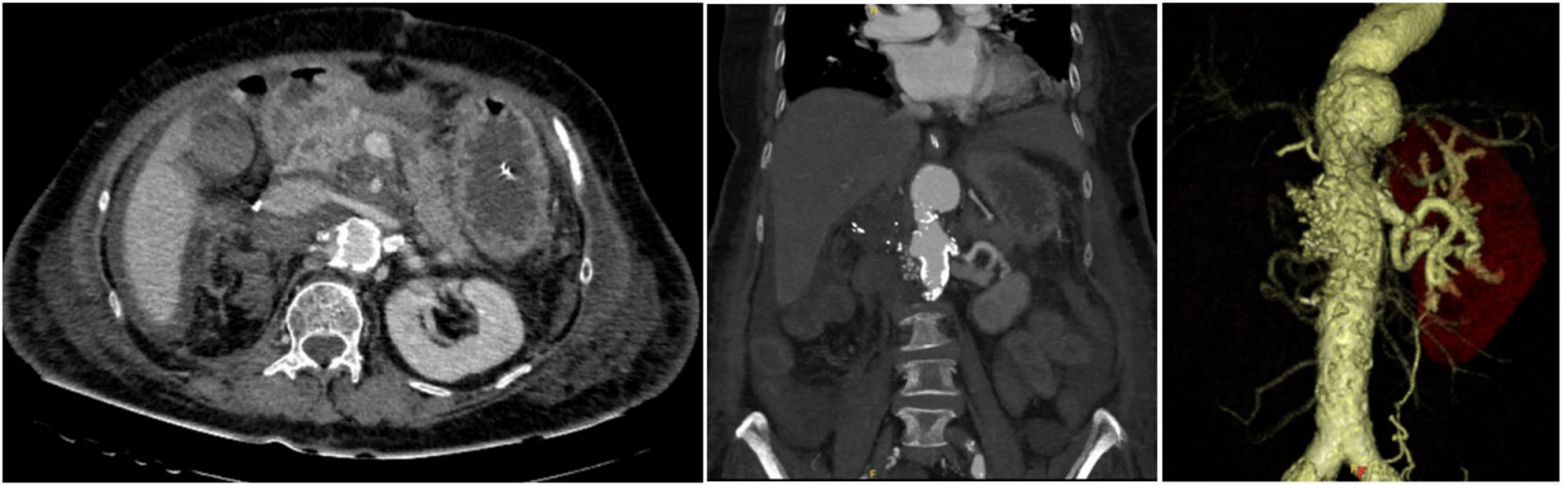
Post-operative computed tomography does not show any leakage around the patch closure. Small granular hyperdensities around the patch should be surgical pledgets and hemostatic material

**Fig. 6:**
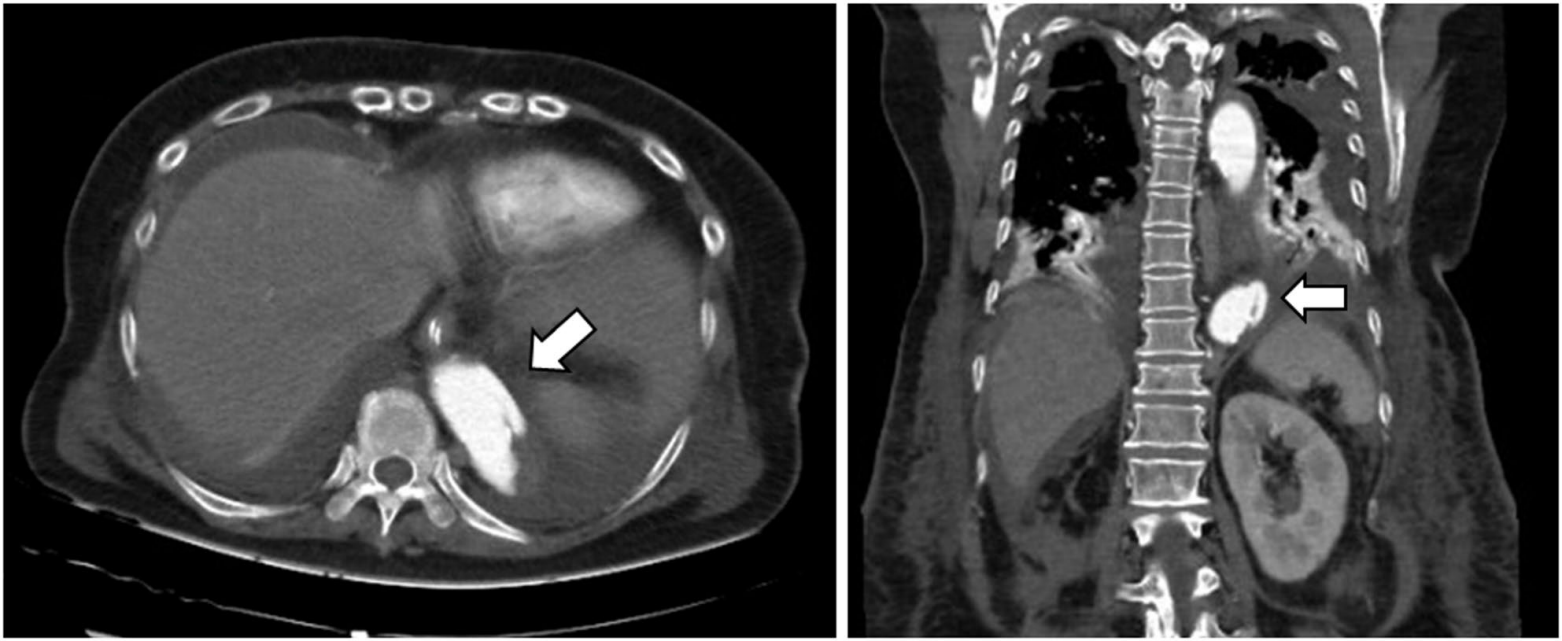
The limited aortic dissection around the balloon clamping site was observed

In case of massive bleeding, a cardiopulmonary bypass was established with right femoral arterial and venous cannulation, considering the potential need for hypothermic circulatory arrest. Two balloon catheters were inserted under fluoroscopic guidance from the left femoral artery and placed in the descending thoracic aorta (RESCUE BALLOON^®^, Tokai Medical Products, Aichi, Japan) and abdominal aorta (Nipro Occlusion Catheter, Nipro, Osaka, Japan), respectively. Simultaneously, a midline incision was made from below the xiphoid process to beneath the umbilicus. We approached the lesion via the right inframesocolic space, placing the pancreas above, to visualize the inferior vena cava and left renal vein. The right RAA was dorsal to the left renal vein (Fig. 3, panel A, B). Dense adhesions around the RAA made clamping of the aorta distal to the superior mesenteric artery challenging. Then, blood flow was occluded by inflating two balloons placed within the thoracic and abdominal aorta respectively. Upon RAA longitudinal incision and thrombus inside the RAA removal (Fig. 3, panel C), a massive bleeding was evidenced. We found the proximal RESCUE BALLOON^®^ placed in the descending thoracic aorta ruptured, so moved Nipro Occlusion Catheter in the abdominal aorta proximally, but it also ruptured. Therefore, another balloon (Pruitt^®^ Aortic Occlusion Catheters, LeMaitre Vascular, Inc., Burlington, USA) was directly inserted in the operative field and deployed in the aorta just proximal to the ostium of the right renal artery. It enabled us to achieve hemostasis. A Hemashield^®^ patch (Getinge, Göteborg, Sweden) closure of the right renal artery inlet was performed (Fig. 3, panel D). The entire abdominal aorta around the aneurysm was highly calcified, disabling the insertion of surgical needles directly through the aorta in some parts. We sutured the caudal part of the patch to the wall of the aneurysm. During this procedure, the Pruitt balloon, which had migrated distally, was damaged by the needle tip and ruptured, so another Pruitt^®^ Aortic Occlusion Catheter was inserted but it also ruptured. We inserted two other balloons (Gore^®^ MOB balloon, W.L. Gore & Associates, Delaware, USA) in the operative field and through the femoral artery, respectively. Figure 4 describes further anatomical relationships and balloon positions in detail. By adding U-stay sutures, we controlled the suture line bleeding around the patch. Although weaning from cardiopulmonary bypass was easy, massive transfusion with 12 units of red blood cells, 8 units of fresh frozen plasma, and 20 units of platelet concentrates was required during the procedure. The aortic clamp time was 90 min, estimated ischemic time for the left kidney was 59 min, and. intestinal ischemia time was estimated at 19 min. The minimum bladder temperature recorded during cardiopulmonary bypass was 34.5 °C. The patient required temporal renal replacement therapy for renal dysfunction postoperatively, however, her general condition was improved in the following weeks. The postoperative enhanced CT revealed no leak around the patch (Fig. 5) but limited aortic dissection around the balloon clamping site (Fig. 6). After 49 days of hospitalization, the patient was transferred to another institution for further rehabilitation.

## Discussion and conclusions

Clamping a severely calcified aorta during cardiovascular surgery poses a significant risk of complications including aortic dissection or rupture, inability to achieve adequate cross-clamping, atherosclerotic embolization, and bleeding [3]. As an alternative, intra-aortic balloon occlusion can be used to avoid the conventional external aortic cross-clamp. However, it also has a high risk of rupture in calcified aortas [4]. Thus, both methods may present difficulties in the control of blood flow required for surgery. Accordingly, preoperative CT imaging is crucial to assess the location and the degree of calcification for adequate surgical approach planning. Generally, when placing an occlusion balloon in the descending aorta, upper limb access provides better stability. However, in this case, both subclavian arteries were severely stenosed or occluded (right 99% stenosis, left complete occlusion). Moreover, maintaining lower-body perfusion via the femoral artery and collecting blood using a pump sucker keep venous return and support physiological antegrade cardiac output, whereas subclavian perfusion could worsen hemorrhage at the rupture site. Therefore, the Pruitt^®^ Occlusion Catheter and then, the Gore^®^ MOB balloon were inserted in the operative field urgently. In contrast to the more flexible shaft of the Pruitt^®^ occlusion catheter, the MOB balloon is characterized by higher shaft rigidity, which proved advantageous for retrograde insertion and hemostasis in large-diameter vessels such as the aorta. However, postoperatively, a localized aortic dissection occurred in the descending aorta, where the occlusion balloon was inflated. In retrospect, we should consider using a rigid guidewire in combination with the MOB balloon. This approach can facilitate straightening the balloon and improve stability minimizing the risk of partial dissection by avoiding excessive inflation pressure.

In some cases where aortic graft replacement is difficult, patch aortoplasty may be another treatment option. This procedure is mainly used to repair coarctation of the aorta in children. However, the suture of the patch can be difficult due to calcification in elderly patients. Moreover, according to a previous report, 20.6% of patients develop patch aneurysms and require reoperation in the remote period [5], and the mean interval from patch placement to aneurysm repair is 15 years [6]. Given these facts, patch aortoplasty can be an option for very elderly patients who are unable to undergo graft replacement surgery.

In high-risk cases, using a cardiopulmonary bypass (CPB) during the surgical procedure is preferable from a risk management perspective. By adjusting the CPB flow, we could maintain a low-pressure state that facilitated precise suturing on the highly calcified and fragile aortic wall. CPB also provided effective volume management and hemodynamic stability during massive intraoperative hemorrhage, which could not have been achieved with standard fluid resuscitation. However, inserting an arterial cannula may be challenging in patients with aortitis, as demonstrated in this case, where our patient had bilateral subclavian artery stenosis and calcification of the right femoral artery. Therefore, thorough preoperative evaluation is crucial to assess vascular accessibility and plan the surgical approach accordingly.

Due to severe calcification of the abdominal aorta and the high-risk location of the aneurysm, we utilized multiple intra-aortic balloons instead of an aortic cross-clamp to achieve successful surgical bleeding control, but it also carries a significant risk of rupture. Successful management requires the proactive establishment of CPB as a safety net for volume recovery and the preparation of multiple, diverse occlusion devices. In patients with severely calcified aortas, occlusion balloons used for EVAR may provide more stable blood flow blockage. Patch aortoplasty is a viable option for elderly patients where graft replacement is impractical.

## Data Availability

Not applicable.

## Abbreviations

CPB: Cardiopulmonary bypass
CT: Computed tomography
EVAR: Endovascular aortic repair
PCI: Percutaneous coronary intervention
RAA: Renal artery aneurysm
